# Opioid, methamphetamine, and polysubstance use: perinatal outcomes for the mother and infant

**DOI:** 10.3389/fped.2023.1305508

**Published:** 2023-12-18

**Authors:** Trecia A. Wouldes, Barry M. Lester

**Affiliations:** ^1^ Department of Psychological Medicine, The University of Auckland, Auckland, New Zealand; ^2^ Center for the Study of Children at Risk, Warren Alpert Medical School, Brown University, Providence, RI, United States

**Keywords:** maternal substance use disorders, opioids, methamphetamine, perinatal outcomes, polysubstance use

## Abstract

The escalation in opioid pain relief (OPR) medications, heroin and fentanyl, has led to an increased use during pregnancy and a public health crisis. Methamphetamine use in women of childbearing age has now eclipsed the use of cocaine and other stimulants globally. Recent reports have shown increases in methamphetamine are selective to opioid use, particularly in rural regions in the US. This report compares the extent of our knowledge of the perinatal outcomes of OPRs, heroin, fentanyl, two long-acting substances used in the treatment of opioid use disorders (buprenorphine and methadone), and methamphetamine. The methodological limitations of the current research are examined, and two important initiatives that will address these limitations are reviewed. Current knowledge of the perinatal effects of short-acting opioids, OPRs, heroin, and fentanyl, is scarce. Most of what we know about the perinatal effects of opioids comes from research on the long-acting opioid agonist drugs used in the treatment of OUDs, methadone and buprenorphine. Both have better perinatal outcomes for the mother and newborn than heroin, but the uptake of these opioid substitution programs is poor (<50%). Current research on perinatal outcomes of methamphetamine is limited to retrospective epidemiological studies, chart reviews, one study from a treatment center in Hawaii, and the US and NZ cross-cultural infant Development, Environment And Lifestyle IDEAL studies. Characteristics of pregnant individuals in both opioid and MA studies were associated with poor maternal health, higher rates of mental illness, trauma, and poverty. Infant outcomes that differed between opioid and MA exposure included variations in neurobehavior at birth which could complicate the diagnosis and treatment of neonatal opioid withdrawal (NOWs). Given the complexity of OUDs in pregnant individuals and the increasing co-use of these opioids with MA, large studies are needed. These studies need to address the many confounders to perinatal outcomes and employ neurodevelopmental markers at birth that can help predict long-term neurodevelopmental outcomes. Two US initiatives that can provide critical research and treatment answers to this public health crisis are the US Environmental influences on Child Health Outcomes (ECHO) program and the Medication for Opioid Use Disorder During Pregnancy Network (MAT-LINK).

## 1. Introduction

The use and misuse of prescription and illicit opioids and amphetamine-type stimulants (ATS) among women of childbearing age has escalated worldwide (1–4). The stimulant that is responsible for the steep escalation in the use of ATS worldwide is crystalline methamphetamine (MA), also known as “ice,” “crystal meth,” “P” in New Zealand (NZ), and “tick” in South Africa (1, 3, 5). Although the global illicit use of these substances during pregnancy is difficult to estimate, three indicators suggest this is a significant public health challenge. The first is the increase in the number of women of childbearing age seeking treatment for substance use disorders or requiring hospitalization due to the abuse of these drugs (5–7). Second is the dramatic increase in rates of neonatal abstinence syndrome (NAS), more recently termed neonatal opioid withdrawal syndrome (NOWS), and adverse perinatal outcomes for the prenatally exposed newborn (1, 3, 8, 9). For example, in the US, from 1999 to 2014, the prenatal use and abuse of opioids, and more recently heroin, resulted in a 333% increase in fetal exposure and a significant increase in NICU admission rates (4, 8, 10). In addition, current estimates of the prevalence of neonatal withdrawal from opioids suggest that in the US, one newborn is diagnosed with NOWs every 18 min (11). Third is the increase in overdose deaths involving methamphetamine and where the use of illicit opioids was reported (12).

The surge in maternal use of opioids was first attributed to the sale of opioid pain relievers (OPRs), with 9.5% to 41.6% filling a prescription in 2007 across 46 states in the United States (US) and 6% in Norway between 2004 and 2007 (13, 14). US national estimates for the total number of opioid prescriptions dispensed also showed a 35% increase between 2000 and 2010. While prevalence data is not available for OPRs during pregnancy in other countries, data from the general population suggest increasing use of prescription opioids in Australia (4), New Zealand (15), Canada (16–18), Germany, Israel, and the United Kingdom (14).

Attempts to curb the opioid epidemic through legislation, education, and the development of abuse-deterrent formulations designed to make inhalation and injection of prescription opioids more difficult meant that abuse of OPRs declined in favor of heroin and synthetic opioids, mainly non-pharmaceutical fentanyl (NPF) (19–21). Misusing these drugs has significantly increased overdose mortality among pregnant and postpartum women and women of childbearing age (21). Using data from the National Vital Statistics mortality files of 7,642 pregnancy-associated deaths between 2017 and 2020, researchers found 1,249 were overdose-related, increasing from 6.56 to 11.85 per 100,000 or a relative increase of 81%. Overdose deaths among women of childbearing age increased from 14.37 to 19.76 per 100,000, a relative increase of 38%. Increased opioid-associated overdose deaths have continued to affect the United States (US) and many developed countries, including Canada, Australia, and countries in the European Union (7). MA overdose deaths during pregnancy have also increased and have been reported to include opioids (7, 12, 20, 22, 23).

In US treatment admissions data reported in the 2008–2017 Treatment Episode Data Set (TEDS), the percentage of primary heroin treatment admissions showed MA use increasing each year from 2.1% in 2008 to 12.4% in 2017, a relative percentage increase of 490% and an annual percent change (APC) of 23.4%. And women of childbearing age had higher percentages of heroin treatment admissions involving MA (2.8% in 2008 to 15.1% in 2017) than males (1.7% in 2008 to 10.8% in 2017). The Survey of Key Informant Patients Program database, which comprises individuals who have entered treatment for an opioid use disorder (OUD) at one of their treatment centers in 49 states and Washington, DC, also reported increased co-use of MA. Of all the non-opioid drugs tracked in this population between 2011 and 2018, MA was the only drug with a significant prevalence increase (85%).

While many people who use licit and illicit psychoactive drugs may prefer a specific drug or drug class, polysubstance use is nearly ubiquitous in people with substance use disorders (SUDs) (23–32). In addition, polysubstance use during pregnancy is only one of several related risk factors. Maternal mental illness, poverty, poor nutrition, involvement with child protective services, and domestic violence are common in women with SUDs (24, 28–30). Understanding the co-use of opioids and MA, and other psychoactive drugs and the context of their use is necessary to prevent maternal morbidity and mortality, inform clinical interventions, and reduce or mitigate adverse perinatal outcomes such as NOWs.

Given the escalation of MA use associated with opioid and opioid use disorders (OUD), the purpose of this narrative review is twofold: first, to briefly review the perinatal effects on mothers and their offspring exposed prenatally to opioids and MA during pregnancy, and second, to explore what we know about the perinatal impact of the co-use of opioids, MA and other commonly used psychoactive drugs, and the social and environmental risk factors associated with maternal SUDs.

## 2. Maternal and perinatal outcomes from prenatal exposure to opioids

Opioids include a wide range of natural and synthetic alkaloid derivates that act as agonists of at least one of the three types of opioid receptors: mu (*μ*), lambda (*δ*), and kappa (*κ*). Drugs from the poppy plant, such as heroin, codeine, and morphine, were initially referred to as “opiates.” Now, the term opiates is often used interchangeably with the term opioid, a more general term that includes natural agonists such as heroin and codeine and synthetic agonists such as fentanyl and oxycodone that, when injected, insufflated, or smoked, enter the brain rapidly and create feelings of pleasure or euphoria, relief from pain, or a state of relaxation or drowsiness.

If more than a medically necessary amount of prescription OPRs such as Oxycontin® and Vicodin® are used, they will have similar pharmacological effects to heroin. Repeated use often results in tolerance to their psychoactive effects and, in turn, dependence to prevent withdrawal symptoms. Opioids like heroin, oxycodone, morphine, and fentanyl are categorized as short-acting opioids or immediate-response opioids. In contrast, oxymorphone hydrochloride extended-release, methadone, and buprenorphine are long-acting or sustained response opioids. Long-acting opioids are mainly prescribed for opioid substitution treatment (OST) but may also be used for pain relief or illicitly for their psychoactive effects.

### 2.1. Maternal outcomes

OUDs during pregnancy are associated with various health and mental health problems. These often depend on the type of opioid (short- or long-acting) and whether the individual is receiving OST. Comorbidities include SUDs of other drugs, chronic pain, HIV, hepatitis C virus, obstetric complications including miscarriage, more terminations, and significant mental health disorders (18, 25, 26, 33). A study (*N* = 174) investigating the relationship of psychiatric symptoms to the severity of drug use and drug-related problems in individuals receiving OST found co-occurring psychiatric symptoms are common and impact the severity of opioid dependence (33). A large percentage (64.6%) of the sample presented with symptoms of a co-occurring psychiatric disorder, 33% with depression, 16% with PTSD, and 39% endorsed hypomania. A large Canadian study comparing methadone maintenance treatment (MMT) with maintenance treatment with buprenorphine (BUP) found 92% of their sample reported mental health disorders (18). A recent study of 21,905 pregnant people in the Environmental Influences on Child Health Outcomes (ECHO) Program reporting opioid use (*N* = 591) found maternal depression was associated with an increased odds of opioid use during pregnancy by more than two-fold (aOR: 2.42, 95% CI: 1.95–3.01) (31).

### 2.2. Perinatal outcomes from prenatal exposure to OPRs

OPRs are prescribed for back and pelvic pain in late pregnancy, which occurs in approximately 68 to 72% of women, and for joint pain, migraine, and myalgia (34). OPRs are associated with an increased prevalence of OUDs; however, studies investigating the perinatal outcomes of the prenatally-exposed infant are sparse (35). One report that reviewed the use of short-acting OPRs, including codeine, tramadol, acetaminophen, oxycodone, and hydrocodone, as well as opioids used for OST (methadone and buprenorphine) during pregnancy, found mixed results in birth outcomes (36). Of the studies examining fetal growth, three studies in this review found no association between low birth weight (<2,500 gms) and oxycodone, codeine, and short-acting opioids overall (37–39). In comparison, one study reported an association between infants born small for gestational age (SGA, <10th percentile) and acetaminophen with oxycodone, codeine, or hydrocodone (11.5% exposed vs. 7.8% unexposed neonates) (40). In contrast, a further study found no association with OPRs but reported an increased rate of large for gestational age (LGA) infants among mothers who used propoxyphene (39).

Preterm birth (<37 weeks) results also varied between studies, with a report from the First Nations population in northwestern Ontario, Canada, reporting a higher percentage of preterm birth in oxycodone-exposed pregnancies (11.5%) compared to nonexposed (7.8%) (38). A Swedish Medical Birth Register study also found a relationship between maternal tramadol use and preterm birth. Still, no association was found in the same study for very preterm births (<32 weeks) (39). Two further investigations found no association with preterm birth, one examining the relationship between codeine use in pregnancy (37) and one where most opioid use was acetaminophen with oxycodone, codeine, or hydrocodone (40).

Case-control and cohort studies have identified a relationship between prenatal exposure to OPRs and congenital heart defects (CHDs), neural tube defects (NTDs), cleft palate, and clubfoot (36, 39, 41). Many of these studies grouped opioids (e.g., different combinations of codeine, hydrocodone, oxycodone, tramadol, and meperidine) and reported on associated congenital disabilities. Of those studies investigating the association between individual opioids and congenital disabilities, more studies found higher odds for a relationship between codeine and CHDs (4 studies) and NTDs (2 studies). Propoxyphene and tramadol had higher odds for clubfoot (36). However, these reviews were published before the dramatic increase in OPRs. Therefore, they did not capture the effects of repeated misuse of these or other prescribed or illicit opioids, psychostimulants, or psychological or lifestyle factors associated with substance use disorders (SUDs) (36, 39, 41).

### 2.3. Perinatal outcomes from prenatal exposure to fentanyl

Fentanyl is a synthetic opioid that is 50 times more potent than heroin and 100 times more potent than morphine. There are two types of fentanyl: pharmaceutical and illicitly-manufactured or non-pharmaceutical fentanyl (NPF) (street names, Apache, Dance Fever, Friend, Goodfellas, Jackpot, and Murder 8). NPF is sold as a powder, dropped onto blotter paper, and put in eye droppers and nasal spray. Some drug dealers are mixing it with cocaine, heroin, MA, and MDMA as a cheap way to boost the psychoactive effects (42). Clinically, fentanyl is used widely in patients undergoing general anesthesia, including women having a variety of surgical procedures throughout pregnancy and for epidurals during labor (43). Human research on the perinatal effects of pharmaceutical fentanyl or NPF is limited. However, a human study and animal models have documented placental transfer to the fetus (44, 45). In a study of 38 women undergoing a termination of pregnancy between 8 and 14 weeks, a rapid transfer of fentanyl to the placenta and the fetal brain was found after an intravenous bolus dose was administered under anesthesia (44). Fentanyl was detected in all 38 placental and all seven of the available brain samples but not in any amniotic fluid. Subsequently, there was a rapid decrease in fentanyl concentrations in maternal serum. However, there was no decline in placental or fetal brain concentrations over the study period (10–30 min), indicating a likely accumulation in the fetus. In animal models, prenatal exposure to fentanyl has been linked to a higher prevalence of newborn mortality, signs of withdrawal, and lasting deficits in sensory processing that extend to adolescence. Impaired sensory processing in children is associated with attention deficit disorder, autistic-like characteristics, schizophrenia, and synesthesia (45).

### 2.4. Perinatal outcomes from prenatal exposure to heroin

Before the 1950s, only a few cases of adverse perinatal outcomes due to prenatal exposure to heroin were reported. In 1956, a review of the literature found ten instances where infants born to mothers dependent on heroin exhibited the characteristic signs of neonatal abstinence syndrome (NAS), including restlessness, yawning, high-pitched cry, tremors, watery stools, hypertonia, seizures, and vomiting (46). An increase in the number of heroin-dependent women presenting to antenatal clinics precipitated further research examining the obstetric and perinatal consequences associated with heroin (47–53). Small numbers, retrospective designs, and selection bias limited the findings in early studies investigating the obstetric and perinatal complications of maternal heroin use. Still, researchers identified some consistent perinatal risks for the mother and her child. Maternal complications were those typically associated with intravenous drug use, such as malnutrition, blood-borne infections (Hepatitis B and C), and skin abscesses. Complications specific to pregnancy included pre-eclampsia, premature rupture of membranes, toxemia, amnionitis, and a high incidence of breech position on delivery.

Perinatal complications for the infant included a high rate of preterm births (28% to 57%), intrauterine growth retardation (IUGR), fetal and neonatal death (3% to 17%), and signs of NAS (8% to 79%). Four studies reported sudden unexplained death in infancy (SUDI) (47, 49, 52, 53), but only two found congenital anomalies greater than the current hospital population (48, 51). Autopsies of 82 infants born to heroin-dependent women between 1954 and 1972 compared to 1,044 consecutive well-preserved stillborn and newborn infants explained the high rate of infant mortality, IUGR, and preterm deliveries (54). Growth retardation was associated with significant reductions in the number of cells in various organs. Almost 60% of heroin-exposed specimens had meconium in the amnion. In several, it was present in the chorion, suggesting fetal distress or withdrawal, resulting in preterm birth or mortality. The mean gestational age of heroin-exposed infants or infants with chorioamnionitis or fevers was 35 ± 3 weeks compared to 39 ± 2 weeks for no observed infection, and heroin-exposed infants who were stillborn or died as newborns also had a high incidence of infection (57%). Notable in the few studies examining the perinatal effects of heroin use in pregnancy was the lack of information about prenatal care, multiple drug use, mental illness, and other lifestyle factors associated with OUDs (55).

More recently, findings from a small randomized controlled trial (RCT) comparing prenatal exposure to heroin, MMT, and BUP found the lowest birthweight, the highest number of newborns with IUGR, and the most numerous placental changes were in heroin-exposed infants. Still, no deaths or congenital abnormalities were reported. However, the severity and course of NOWS were the poorest for infants born to mothers receiving MMT (56).

### 2.5. Maternal and perinatal outcomes from OST with methadone and buprenorphine

With the introduction of MMT in the 1970s as an opioid substitution treatment, many of the adverse outcomes associated with the maternal use of “street heroin,” such as malnutrition, anemia, blood-borne illnesses from shared needles (Hepatitis C and HIV), and obstetric complications were mitigated (57–59). Advantages of MMT included stabilization of opioid levels, reduced illicit drug use, criminal activity, maternal mortality, and improved engagement with healthcare (60–62). Improved clinical outcomes at birth for infants whose mothers were receiving MMT compared to heroin were also reported (60, 63–67). MMT-exposed infants weighed significantly more than heroin-exposed infants and infant mortality was reduced. However, several studies have shown methadone crosses the placenta, affecting fetal motor activity, breathing movements, heart rate, and parasympathetic tone due to altered fetal neurodevelopment (68–71). Infants are at increased risk of being born early and, when born at term, to be symmetrically smaller (weigh less, be shorter, with smaller head circumferences) than infants born to mothers using multiple non-opioid drugs (25, 26, 72–74). The risk of SUDI strabismus, nystagmus, and hyaline membrane disease is also greater for MMT-exposed infants compared to non-opioid exposed infants (25, 75–79). More recently, research has shown that BUP may provide better clinical outcomes for neonates (18, 80–84).

Methadone is a synthetic full opioid agonist that primarily activates the µ-opioid receptor and the κ- and δ-opioid receptors. These are widely distributed across the CNS and peripheral and gastrointestinal systems (85). Its psychoactive effect is mild euphoria but also results in respiratory depression. In comparison, buprenorphine is a partial μ-opioid agonist and *κ*-opioid antagonist that produces similar morphine-like psychoactive effects at a relatively lower dose. However, these effects are weaker than full opioid agonists. At higher doses, buprenorphine has a “ceiling effect” where higher doses are associated with much smaller increases in the psychoactive effects and less respiratory depression, reducing the risk of abuse and accidental overdose (86).

The effects of BUP and MMT on the developing nervous system are evident in fetal behavior and infant clinical and neurobehavioral outcomes (26, 68, 70, 87–89). Two reports of participants enrolled in the Maternal Opioid Treatment: Human Experimental Research (MOTHER) study compared indices of fetal neurobehaviour in BUP-exposed fetuses to MMT-exposed before and after dosing with buprenorphine or methadone (90, 91). The first, a pilot study (*N* = 3 BUP vs. 3 MMT) at two-time points in gestation (24–28 and 32–36 weeks), found BUP was associated with higher levels of FHR variability, more accelerations and greater fetal movement-FHR coupling as well as a trend towards longer movement duration at the earlier gestation period (91). No differences in cardiac measures were found later in gestation, but overall motor activity was significantly depressed in the MMT-exposed fetuses (91). The second study compared BUP- and MMT-exposed fetuses at 31–32 weeks gestation (*N* = 33 BUP vs. *N* = 48 MMT). No group differences were found in FHR or FHR accelerations, but there was a significant decrease in FHR accelerations from pre- to post-dose in the MMT group. A non-reactive stress test occurred more frequently in the MMT group overall. However, depressed fetal movement was observed in both groups post-dose (90). More recently, depressed FHR, fewer heart rate accelerations, and depressed fetal movements were observed 2.5 h post-dose in BUP-exposed pregnancies at 24, 28, 32, and 36 weeks gestation. The magnitude of these effects increased across gestation (87).

### 2.6. Clinical outcomes at birth from prenatal exposure to MMT and BUP

Of the studies that have compared BUP- to MMT-exposed infants, some have found no differences in the risks of fetal death, preterm birth, low birth weight, and SGA/growth restriction (56, 92, 93), while others have reported a lower risk of preterm birth and higher birth weights for BUP-exposed compared to MMT-exposed infants (94, 95).

A particular focus of outcomes at birth has been the incidence of NAS or NOWs and, more recently, the neurobehavior in children born to mothers receiving MMT or BUP (80, 86, 96–102). The percentage of children exposed to MMT with any signs of NAS varies between 24% and 100%, with 54%–85% requiring pharmacological treatment to alleviate the severity of withdrawal symptoms (96, 101, 102). Several international studies comparing MMT with BUP show NAS is equally common among children born to mothers receiving BUP, occurring in approximately 40%–90% of exposed neonates, with a similar proportion requiring pharmacotherapy (50%) (83, 92, 93, 95, 103–105). In comparison, several studies have found MMT-exposed infants required higher doses of opioid agonist medication to treat NAS than BUP (83, 105, 106) and were more likely to spend more time in the hospital postnatally (83, 104–106). The variability in NOWS may be associated with differences in the clinical assessment and management of these infants postnatally, opioid type, and daily dose. For instance, larger maternal methadone doses in pregnancy have been associated with an increased risk of withdrawal (79, 107–112), but other studies found no relationship (101, 113–117). Other factors associated with the risk of NOWS are exposure to other substances, including stimulants, barbiturates, nicotine, and SSRIs (9, 18, 96) and preterm birth. Preterm infants exhibited fewer signs of withdrawal and a less severe or prolonged course of symptoms (109, 112, 118). Finally, a recent study found the duration of stay in hospital and the need for pharmacological treatment were related to variants in the *OPRM1* and *COMT* genes (119).

### 2.7. Neurobehavior at birth from prenatal exposure to MMT and BUP

Infant adaptation to the postnatal environment is essential for promoting organized patterns of feeding and sleep and in the early development of the parent-infant relationship (120). Neurobehavioral studies using the Brazelton Neonatal Assessment Scale (NBAS) and the Neonatal Intensive Care Unit Network Neurobehaviour Scale (NNNS) have found neurobehavioral differences between OST-exposed and nonexposed infants (26, 88, 89, 120, 121). The NNNS is a well-validated neurobehavioral scale explicitly designed for detecting neurological and behavioral function and stress abstinence in the drug-exposed infant at birth (123). Two US studies compared MMT-exposed infants requiring pharmacotherapy for NAS with those who did not (88, 89). One compared MMT-exposed to a published normative sample of healthy, unexposed infants (88, 124). A NZ study compared MMT-exposed infants at birth with a nonexposed group in the prospective, longitudinal Methadone in Pregnancy Study (MIPS) (26). All studies found MMT-exposed infants had a more dysregulated pattern of neurobehaviour at birth than unexposed infants. Significant differences were found in habituation scores, attention, handling, non-optimal reflexes, hypertonicity, hypotonicity, and stress abstinence. A small study compared MMT-exposed (*N* = 21) neurobehaviour with BUP-exposed (*N* = 16) infants on days 3, 5, 7, 10, 14–15, and 28–30 days postpartum. The neurobehavior of both MMT and BUP-exposed infants improved over time. Still, infants exposed to BUP *in utero* exhibited fewer stress-abstinence signs, less hypertonia, better self-regulation, and required less handling (122).

Several studies have suggested the improved outcomes for BUP over MMT may be due to the differences in social or lifestyle factors and psychological or substance use problems between those prescribed BUP compared to those prescribed MMT during pregnancy. For instance, significantly more mothers randomized to buprenorphine treatment in RCTs have dropped out of studies, reportedly because of dissatisfaction with the study medication (83, 92, 125). Additionally, in cohort studies, buprenorphine was more likely to be prescribed to women with less serious social and substance dependence problems and more stable lifestyles (103, 126–128). In the MOTHER RCT, women randomized to buprenorphine were likelier to have less prior opioid use (125). Still, a recent cohort study involving pregnant persons enrolled in a public insurance program in the US (*N* = 2,548,372) from 2000 to 2018 found no association between the above differences and perinatal outcomes (84). Analyses adjusted for several factors associated with OUDs found NAS occurred in 52% of infants exposed to BUP compared with 69.2% exposed to MMT. Preterm birth occurred in 14.4% of infants exposed to BUP and 24.9% to MMT. SGA (12.1% vs. 15.3%) and LBW (8.3% vs. 14.9%) were less prevalent in BUP-exposed infants, respectively. Still, the risk of adverse maternal outcomes was similar between BUP- and MMT-exposed persons.

Although several studies report more favorable neonatal outcomes for BUP than MMT (84), both can be safely used in pregnancy and are recommended over untreated OUDs. Illicit use of short-acting opioids such as heroin and fentanyl exposes the mother and fetus to dangerous fluctuations in blood morphine levels, unknown drugs and contaminants, and infections such as hepatitis B and C and HIV with the potential for severe morbidity and mortality for the mother and her infant (94, 104, 106, 129, 130). Still, reports show that, on average, less than 50% of pregnant individuals with OUDs are receiving OST (18, 131), and discontinuation of MMT was reported to be higher for individuals who reported weekly use of MA (132).

## 3. Maternal and perinatal outcomes from prenatal exposure to methamphetamine

### 3.1. Maternal outcomes

MA is classed as a psychostimulant, chemically similar to amphetamine but with significantly more potential for harm due to its higher potency and longer half-life (10–12 h). MA is a vasoconstrictor, decreasing blood flow leading to hypoxia (133, 134). Its effects are mediated through the release of dopamine, serotonin, and norepinephrine and blockage of intracellular vesicular monoamine transporter 2 activity. Its psychoactive effects are euphoria, increased alertness, libido, a feeling of extreme well-being, and decreased appetite (1). Withdrawal symptoms are fatigue, drowsiness, and depression (135). Craving may start within a few hours and last for two weeks. Tolerance to MA is rapid, leading to “telescoping” of use where more MA and shortened duration of use is required to maintain the desired psychoactive effects. The pattern of use is episodes of bingeing that can last for two weeks (136). The longer half-life and broader target sites of MA in the CNS mean there may be more severe outcomes for the exposed mother, the fetus, and the developing child than from other stimulants (137).

Consistent with the current evidence on the impact of OPRs and short-acting opioids in pregnancy, most existing studies on prenatal MA use tend to focus on the prevalence of prenatal exposure rather than the perinatal outcomes for the mother (7). In one US study, a high percentage of women who used MA were found to have early pregnancy loss (41%) before 26 weeks gestation, which is twice the National average (137). Yet, no indication of whether this loss was due to miscarriage or termination was reported. A further study showed that 33% of pregnancies in women who use MA end in termination of pregnancy, compared to 18% in the general population in the US (137). A large retrospective study in the US found amphetamine-affected births had the highest rates of pre-eclampsia (9.3% vs. 4.4% opioid, 4.8% other), cesarean delivery (37.4% vs. 34.5% opioid, 32.6% other), placental abruption (4.3% vs. 3.1% opioid, 1.0% other), preterm delivery, <37 weeks (16.7%, vs. 12.6% opioid, 5.8% other), and severe maternal morbidity or mortality (2.9% vs. 2.4% opioid, 1.6% other) (138).

MA use during pregnancy is also associated with a higher risk of cardiovascular disease (CVD) (139). A report investigating CVD in women with delivery hospitalizations between 2004 and 2018 in a Nationwide Inpatient Sample showed substance use (SU) was associated with several risk factors related to CVD (139). The prevalence of CV risk factors increased across the study period and included obesity, chronic hypertension, pregestational diabetes, tobacco use, and hyperlipidemia. A total of 60,014,368 delivery hospitalizations occurred, with SU complicating 955,531 deliveries (1.6%). Substances of interest were cocaine, alcohol, cannabis, amphetamine/methamphetamine, polysubstance use, and opioids. Adjusting for demographics, risk factors, and pre-existing conditions, SU use was independently associated with CV events (aOR: 1.61; 95% CI: 1.53–1.70), major adverse cardiac events (aOR: 1.53; 95% CI: 1.46–1.61), and maternal mortality (aOR: 2.65; 95% CI: 2.15–3.12). All substances were associated with an increased risk of acute CV events. However, the risk was most significant in those deliveries with documented amphetamine/methamphetamine use, including a 9-fold increased risk of acute cardiomyopathy or heart failure (aOR: 9.06; 95% CI: 7.52–10.93), acute myocardial infarction (aOR: 7.57; 95% CI: 4.12–13.92), cardiac arrest (aOR: 7.29; 95% CI: 4.19–12.68), and maternal mortality (aOR: 3.20; 95% CI: 1.59–6.41). Opioid use had the strongest association with endocarditis, alcohol use had the strongest association with arrhythmias, and cocaine had the strongest association with stroke. All substances were strongly associated with maternal mortality and major adverse cardiac events, except cocaine and cannabis, which were related to increased maternal mortality.

Consistent with reports on OUDs, prenatal MA use was associated with maternal mental illness, increased reports of domestic violence, poverty, and maternal histories of physical or sexual abuse in the cross-cultural multisite US and NZ Infant Development, Environment And Lifestyle (IDEAL) studies (24, 140). MA use in both the US and NZ studies was associated with being single, waiting longer to attend the first prenatal visit, being more likely to have child protection (CPS) referrals, and using several other drugs than a matched comparison group. MA use in the US study was associated with less prenatal care than the US comparison group and less adequate prenatal care than MA use in the NZ study. Additionally, inadequate prenatal care in the US was associated with increased child protection referrals related to MA use. In contrast, referral to CPS in NZ required more serious social issues related to child safety other than MA use (140). A comparison of maternal mental illness in the US and NZ IDEAL study found MA use was associated with more symptoms associated with paranoid ideation, depression, and interpersonal sensitivity. US (*N* = 127) and NZ (*N* = 97) mothers who used MA were 10 times more likely than their respective matched comparison group (US *N* = 193. NZ *N* = 110) to have an SUD and twice as likely to meet the criteria for a psychiatric disorder. In NZ, but not the US, women who used MA in pregnancy had a significantly heightened risk (five-fold) for comorbid SUD and a positive diagnosis for a psychiatric disorder. This disparity may be due to higher quantities of alcohol use in the NZ sample than in the US. In addition, up to 31% of individuals using MA enrolled in the US and NZ IDEAL studies self-reported continued psychiatric comorbidities one month after birth (141, 142).

### 3.2. Clinical outcomes at birth from prenatal exposure to MA

Early studies of prenatal exposure to MA found associations with an increased incidence of cardiac defects, cleft lip, biliary atresia, stillbirth, cerebral hemorrhage, Mongolian spots, systolic murmur, and undescended testes (143). Adverse somatic growth effects were also reported (144, 145). Yet, these reports were reliant on chart review, were retrospective, had small samples, and lacked adjustment for the confounding factors associated with maternal SUD, such as mental health, other drug use, and poverty. A Swedish longitudinal study found female infants exposed to MA were lighter and shorter, but there was no difference between exposed and nonexposed males (146). They also reported that exposed infants were more likely to be drowsy in the first postnatal months (147). Their study, however, lacked a matched comparison group, had a small sample (*N* = 65), and included other drugs along with amphetamine. Also, as this study began in 1980, it is unlikely that MA-exposure was the primary amphetamine used in these studies.

No differences between MA and comparison groups in the incidence of facial dysmorphism, skeletal or cardiac defects, or respiratory problems were observed in the IDEAL Study at birth (142). Admission to the NICU was higher for the MA-exposed infant, and after adjusting for covariates, MA exposure remained significantly associated with poor suck and less likely to be breastfed. No difference between MA and nonexposed comparisons was observed for central nervous system signs of drug withdrawal, and none of the infants required pharmacological interventions.

Studies examining the growth of MA-exposed infants have found, after adjusting for covariates, lower birth weights, smaller head circumferences, and shorter length at birth (148–150). In one study, infants with positive toxicology (meconium) for MA at birth were smaller than infants with first-trimester exposure only (2,932 g v. 3,300 g, *P* = 0.01) and compared to nonexposed infants were born significantly earlier (37.3 weeks vs. 39.1, *P* = 0.0002). Those women in this report who stopped using MA during pregnancy had normal births (148).

The impact of prenatal exposure to MA on growth in the US vs. the NZ IDEAL cohorts found a stronger negative effect on infant and child length/height in the US (151). NZ has a harm reduction policy around maternal drug use and provides free prenatal and postpartum care for all. These findings suggest that improved antenatal care for mothers with a SUD can potentially prevent decreased growth observed in the US (152).

Examination of neurobehavior at birth using the NNNS found MA-exposed infants in the US and NZ samples exhibited poorer quality of movement and increased physiological stress, total stress/abstinence, and CNS stress (153). Additionally, infants with heavy MA exposure exhibited lower arousal and less excitability when compared with nonexposed infants. These findings from the US and NZ increase the generalizability of MA exposure across cultures.

## 4. Polysubstance use and other risk factors associated with OUDs

### 4.1. Diagnosing NOWS in the context of polysubstance use during pregnancy

Models of cumulative risk would suggest that there is a continuum of impairment in perinatal outcomes where there is prenatal exposure to multiple drugs compared to a single drug (154). For instance, there were significant differences in fetal neurobehaviour, NAS, and preterm birth in a study comparing maternal exposure to MMT alone (MMT/A), MMT plus polysubstance use (MMT/P), and no MMT or drug exposure (NMP) (155). Substance exposure in the MMT/P group, in addition to methadone, included cocaine, benzodiazepines, barbituates, cannabis, and non-methadone opioids. MMT/P exposure was associated with acute suppression of fetal breathing and body movements (155), with evidence of a continuum of impairment in fetal heart rate (FHR) and FHR variability. At peak levels of methadone exposure, FHR and FHR variability were significantly decreased in the MMT/P group compared to the MMT/A and NMP groups. Neonatal differences were found between the MM/P and MMT/A group, with the former group being born on average one week earlier and twice as many requiring pharmacotherapy to treat NAS (83.3% vs. 42.1%).

More recently, a large population study of mothers with OUD who were receiving opioid agonist treatment (OST) with either MMT (*N* = 26,740), BUP (*N* = 211), or slow-release opioid morphine injectable agonist treatment (SROM) (*N* = 19), found a high prevalence during pregnancy of other non-opioid and non-alcohol substance use disorders (SUD) (92%) (18). Co-prescription of SSRIs, benzodiazepines, antipsychotics, or the use of stimulants increased the odds of preterm birth [1.6 (95% CI: 1.2–2.1)] and disorders related to SGA or low birth weight [1.4 (95% CI: 1.1–1.8)] after adjusting for treatment duration (18). Over 90% of the women in the study population were diagnosed with a mental health disorder before delivery, with 37% receiving prescribed psychotropic medications during pregnancy.

Polysubstance use may provide a synergistic effect when two drugs are used together, or individual drugs may counteract or modify the perinatal effects of another drug. For instance, while several drugs can cause NAS on their own, co-exposure with opioids can cause differing signs of withdrawal and short- and long-term outcomes and alter withdrawal severity, duration, and timing (156–159). Co-use of benzodiazepines and other psychotropics, such as SSRIs and gabapentin, have been reported in studies of opioid use during pregnancy, predominantly with MMT or BUP. Evidence of their co-use during pregnancy is known to increase the severity, duration, and onset of withdrawal (156, 158, 159). A study of 822 confirmed cases of NAS found infants exposed antenatally to benzodiazepines had greater than 50% increased odds of developing pharmacologically treated NAS (*N* = 598, 72.7%) than a group not requiring pharmacological treatment (*N* = 224). Both treated and non-treated groups had similar exposures to tobacco, tetrahydrocannabinol (THC), cocaine, MA, phencyclidine (PCP), selective serotonin reuptake inhibitors (SSRIs), tricyclic antidepressants, and gabapentin (158).

Increased use of gabapentin with methadone has also been reported during pregnancy with atypical signs of withdrawal, including tongue thrusting, nystagmus, excessive arching of the back, and exaggerated myoclonic jerks (160). Gabapentin is usually prescribed to treat partial seizures, neuropathic pain, and restless leg syndrome. Of a survey of 129 respondents who were using non-prescribed gabapentin, 22% reported using gabapentin in conjunction with methadone, with 38% of those citing gabapentin's ability to potentiate the “high” of methadone as their reason for the concurrent use (161).

Finally, a report investigating the effects of polysubstance use on length of treatment and length of stay for prenatal opioid exposure found similar outcomes between infants exposed to opioids alone (*N* = 33, 19%) or with polysubstance use (*N* = 142. (81%), suggesting opioids were the main driver of hospital outcomes (162). However, a higher percentage of infants with both short- and long-acting opioid exposure required pharmacologic treatment compared to either opioid alone. Results comparing short-acting and long-acting opioids found short-acting opioids decreased the length of treatment. In contrast, long-acting opioids increased the length of treatment, length of stay, and the need for adjunctive therapy. Notably, coexposure of opioids with stimulants decreased the length of treatment and reduced the need for adjunctive treatment. As short-acting opioids were shown to reduce the length of treatment, this observation may reflect the properties of short-acting opioids rather than exposure to stimulants.

### 4.2. The context and risk factors of perinatal outcomes in OUDs during pregnancy

Understanding the characteristics of individuals and the risk factors associated with OUDs during pregnancy has policy, treatment, and clinical implications. Data from the US Environmental Influences on Child Health Outcomes (ECHO) Program obtained characteristics of 21,905 pregnancies that occurred between 1,990 and 2021 (31). Participants who used opioids during pregnancy were more likely to be non-Hispanic White (67%), have a lower socioeconomic status, and 69% reported some college education. Opioid use was present in 2.8% (*N* = 591) of pregnancies. Opioid use, compared to non-use, was associated with high rates of alcohol use (32% vs. 19%), tobacco use (39% vs. 11%), marijuana use (16% vs. 5%), and illegal drugs (10% vs. 1%). Stimulant (MA and cocaine) use was also significantly higher in those pregnancies where opioid use was reported. Only 5% reported heroin use, and 86% of opioid use originated from a prescription. After adjustment for socioeconomic factors, comorbidities, and prenatal use of other substances, only prenatal use of tobacco and any illegal drugs were associated with higher odds of prenatal opioid use. In addition, maternal depression was associated with a two-fold increase in opioid-exposed pregnancies (aOR = 2.42, 95% CI: 1.95–3.01).

A retrospective review of a nationally representative sample of hospital discharges in the US using data from 2014 to 2015 compared birth outcomes and polysubstance use between ATS-affected (*N* = 18,050), opioid-affected, and (*N* = 50, 011) other hospital births (*N* = 7,545,380) (138). A higher percentage of participants in both ATS and opioid use groups had Medicaid as the primary payer, resided in rural counties (ATS 21.5%, opioid 21.7% vs. other 13.3%), and lived in areas where there is the poorest national income quartile compared with other deliveries. Perinatal outcomes were adjusted for age, payer, income, rural vs. urban, and hospital region. Comorbid tobacco use was reported in approximately half of the deliveries of ATS- and opioid-affected pregnancies (46% and 55%, respectively) compared to other hospital deliveries (5.1%). Polysubstance use was more prevalent in ATS- and opioid-exposed pregnancies overall. However, cannabis (26.4% vs. 10.4%) and alcohol (5.1% vs. 1.9%) use were significantly higher in ATS-exposed pregnancies than in opioid-exposed pregnancies. And in 12.6% of ATS-exposed deliveries, co-use of opioids was identified.

A large US representative sample of pregnant women with an OUD living in urban (*N* = 81,515) and rural (*N* = 25,545) regions found the rate of polysubstance use varied by region and drug used (163). The rate of polysubstance use diagnosis among women with OUD at delivery increased more among those women residing in rural (13.8% increase) compared with urban counties (3.5% increase). Diagnosed use of ATS and OUD nearly doubled among those living in rural (255.4% increase) compared to urban counties (150.7% increase). Equally, tobacco use and OUD increased in rural (30.4% increase) more than in urban (23.2% increase) regions. Whereas diagnosed use of cocaine and OUD declined significantly in rural (70.5% decline) and urban (61.9% decline) counties**.**

The characteristics of the population who are pregnant with an OUD or using MA are from lower socio-economic areas. Currently, increased use of OUD and MA are reported in rural counties in the US compared to urban areas, which has implications for whether specialized prenatal and maternity services exist in these areas. In addition to poverty, maternal health, trauma, domestic violence, mental illness, and CPS involvement (140).

## 5. Discussion

This report highlights the significant increase in the use of opioids and stimulants in pregnancy, along with a constellation of other drugs (31, 138, 163). The opioids that are currently associated with prenatal use are immediate reward or short-acting OPRs, heroin or non-prescribed fentanyl (NPF), or illicitly manufactured fentanyl. The perinatal effects of these are largely unknown. Long-acting or sustained-release drugs, methadone and buprenorphine, which are predominantly used in the treatment of OUDs, have received the most attention in the extant literature. Maintenance programs using these drugs show improved maternal health and perinatal outcomes for opioid exposure. Despite the availability of MMT and BUP programs to treat OUDs during pregnancy, less than 50% of pregnant individuals and individuals of childbearing age with an OUD are enrolled in these. The lack of uptake of these programs is likely due to the many barriers to reporting SU and engaging with the health care system that need to be addressed, particularly for women and other already marginalized populations (164–168). These populations may under-report their SU due to the stigma of drug use, lower socioeconomic status, racism, involvement with the criminal justice system, and the threat of child custody proceedings. Women are less likely to seek treatment when there is no accommodation to accept their children or specialist services are lacking, particularly in rural regions (169).

Notable is the finding that opioid use has shown a parallel increase in SUDs associated with MA. And MA use has now eclipsed the use of cocaine and other stimulants globally in women of childbearing age (1, 131). Our review of the perinatal outcomes for individuals with OUDs compared to individuals reporting SUDs associated with MA shows higher rates of severe morbidity and mortality with MA use. MA is associated with significantly higher rates of pre-eclampsia, cesarean delivery, placental abruption, and preterm birth than opioids and other drugs (138). CV events during hospitalizations for delivery are also significantly higher in MA-exposed pregnancies than opioid-, cocaine-, alcohol- or cannabis-exposed pregnancies, including a 9-fold risk for cardiomyopathy or heart failure (139). Still, little is known about the ongoing physical health of those mothers who may be using a combination of opioids, MA, and other drugs. Mental illness, poverty, domestic violence, homelessness, and food insecurity occur frequently in pregnant individuals with substance use disorders. Yet, the complexity of these circumstances has made it difficult to determine the impact of these on the ability to parent an already vulnerable child exposed prenatally to opioids and MA.

This review has shown differences in the neurobehavioral outcomes between opioid-exposed and MA-exposed infants. What is unclear is the effect that using both of these drugs will have on perinatal outcomes and the management of these infants in the context of polysubstance use. For instance, Polysubstance use is ubiquitous and, depending on the type or class of drug, may impair fetal neurodevelopment, increase the need, duration, and adjunctive treatment for NOWs, or suppress or change the signs typically associated with NOWs (156–158, 160, 161). Lacking in many studies is the ability to determine the frequency of use or dose of a particular drug or drugs. Biological measures are often limited to detecting prenatal drug exposure after 20 weeks gestation but not during preconception, embryogenesis, or the first trimesters. In addition, they can not tell us the frequency or pattern of prenatal exposure (164). This is of particular importance in determining the extent of short-acting drugs or MA or the range of new psychoactive substances that continue to emerge in the illicit drug market (131). The pattern of MA use is often bingeing that lasts for weeks, where significant amounts of tobacco, cannabis, and alcohol are consumed. Although self-report measures are limited by recall, combined with biological measures, they may provide a better estimate of the extent of prenatal drug exposure to the mother and newborn (164).

Knowing which drugs have been used prenatally, their frequency, and timing also affect clinical decision-making during the perinatal period. In mothers who are receiving OST, breastfeeding is encouraged as small amounts of opioids in breast milk may moderate signs or severity of NOWs. However, the evidence for breastfeeding infants exposed to MA is less clear. Recommendations for small amounts of MA early in pregnancy suggest the benefits of breastfeeding outweigh the risks of MA exposure. However, breastfeeding is not recommended if there is long-term use or use in the third trimester (170, 171). Therefore, mothers may need to be counseled on alternative ways of feeding or providing breast milk to their infants if there is co-use of substantial amounts of MA use with opioids.

Additionally, infants exposed to MA prenatally have not displayed the typical signs of NOWS (153). Neurobehavioral assessments using the NNNS have found differences between opioid-exposed and MA-exposed infants, with MA-exposed infants exhibiting lower arousal and less excitability (26, 153). Again, the co-use of opioids and MA may depress or exacerbate the effects of opioids and impact the assessment and diagnosis of NOWs (162).

### 5.1. Diagnosing and treating NOWs

A further limitation of current research is the need for more consensus around the best method of assessing and diagnosing NOWs when physiological signs are atypical due to exposure to a combination of different substances (156). In these cases, the decision to use pharmacological or non-pharmacological interventions is left to the clinician. The gold standard for assessing NOWs is the Finnegan Neonatal Abstinence Score Sheet (172), but some researchers and clinicians have conveyed concerns about its subjectivity, length, reliability, and validity (173). Typically, the signs of opioid withdrawal include evidence of some or all of the following: central nervous system (CNS) irritability, high-pitched continuous crying, decreased sleep, increased muscle tone, hyperactive Moro reflex and potential seizures, gastrointestinal dysfunction, feeding difficulties, and vomiting, and autonomic nervous system activation that includes fever, sweating increased respiratory rate and nasal stuffiness and flaring (174).

Recently, a newer function-based—Eat, Sleep, Console (ESC) care—approach was proposed (175). The ESC waives the identification of these typical signs and symptoms unique to each infant and their impact on dyadic functioning and neurodevelopment. Instead, the focus of ESC is evaluating infants in 3 functional capacities: the ability to eat (infant able to eat >1 oz per feed or breastfeed well), sleep (sleeps undisturbed for ≥1 h), and be consoled from crying within 10 min. The reported goal of this method of identifying and treating NOWs was shorter length of stay, reduced medication, and lower costs, all of which are important goals (175). However, concerns have been raised about the widespread use of this tool before it was subjected to randomized controlled trials compared to traditional care (176). Early concerns were the minimized appreciation of the complex neurobehavior that occurs at birth that is disrupted by NOWs when the focus of treatment is only 3 areas of function in the newborn. And the unstated lack of concern for the importance of typical and atypical neurobehavior to later neurodevelopment.

Subsequent research has provided evidence that ESC meets its intended goals in a multisite study of 26 US hospitals. When it was compared to usual care no predetermined adverse outcomes were observed (177). These included seizures or accidental trauma, respiratory insufficiency related to opioid therapy, or a composite safety measure through 3 months of age that included acute or urgent or emergency department visits or hospital readmission. Still, ESC discounts the usefulness of identifying typical and atypical neurobehavior exhibited in multiple systems of infants prenatally exposed to opioids and other substances limits our ability to understand the linkages between NOWs and later neurodevelopment (178).

Finally, few studies examine how the timing of opioid exposure and other substances can impact neurodevelopmental outcomes, nor have the research accounted for the potential confounding of the genetic makeup of the parents or epigenetic factors associated with addiction.

### 5.2. Addressing the limitations and gaps in our knowledge

To address the many limitations of the extant literature and the long-term effects of prenatal exposure to opioids, MA, and other licit and illicit substances, we first need to design large studies that can address the many confounders associated with OUDs and other SUDs. Second, we need to develop evidence-based assessments that will improve the diagnosis and management of prenatal exposure to opioids, MA, and other drugs. This means we need to assess every infant with atypical signs or symptoms associated with maternal drug use. To do this, studies should include a short-term marker of neurodevelopment as a marker for risk for later neurodevelopmental impairment in infancy and childhood (178). For example, this review has shown the differences at birth in neurobehavior between mothers receiving MMT and BUP during pregnancy. We have also demonstrated the differences in neurobehavioral signs in MA-exposed compared to opioid-exposed infants using a standardized measure, the NNNS. Studies employing the NNNS have shown this measure can be used to measure neurodevelopment at birth and is predictive of cognitive and motor development at 24 months (26) and low IQ, adaptive behavior, and problem behavior in 4.5-year-old children (179).

One research program that will be able to address the many limitations of the current studies on SUD during pregnancy is the US Environmental Influences on Child Health Outcomes (ECHO) program, which was initiated in 2016 to examine how environmental exposures in early life can impact health across the life-course (180). This study is designed to identify the mechanisms and intervention targets to address 5 pediatric health outcomes: prenatal, perinatal, and early postnatal outcomes; childhood obesity; airways; neurodevelopment; and positive health outcomes. The Person Reported Outcomes (PRO) Core is a key component of the ECHO program. This unifying measurement framework takes a lifespan development approach to assess how physical, mental, and social health interact within families across the life course to promote or hinder child health outcomes (181). Recent evidence from the ECHO program reported in this review provided the characteristics of 21,905 pregnant individuals who used opioids during pregnancy (31). For a comprehensive review of how the ECHO program can address the methodologic limitations associated with the current literature on maternal OUD and other SUDs, see Condradt et al. (178).

A further important initiative that will inform the treatment of maternal OUDs is the Maternal and Infant Network to Understand Outcomes Associated with Use of Medication for Opioid Use Disorders During Pregnancy or MAT-LINK (182, 183). This project is a surveillance system that examines the demographic characteristics and clinical information of pregnant persons receiving medication for OUDs (MOUD) compared to those who are not receiving treatment. This initiative aims to understand better the effect of treatment outcomes and, in turn, inform public health and clinical care for this population. Data collected in this longitudinal project includes outcomes at delivery and short- and long-term outcomes for children, including physical growth and development, diagnoses of chronic conditions, health care use, vaccinations, and neurodevelopmental outcomes. Given the lack of uptake of BUP and MMT programs (<50%), this initiative will likely provide evidence to improve enrolments in OST programs.

## 6. Conclusions

The conclusions that can be derived from the current literature regarding the perinatal outcomes of the combined increase in prenatal opioid and MA exposure are limited due to the lack of current research and the methodological limitations of available research. Illicit drug use during pregnancy has spiraled out of control since the 1970s, and research on its effects has struggled to keep up. Most studies have focused on the drug “crisis” of the moment. Therefore, many studies are retrospective or epidemiological and can tell us we have a problem but not how to address it. Many of the limitations of the current research have been addressed by the ECHO and MAT-LINK studies, but more studies that address the limitations of past research are needed. To engage participation in research and increase enrolment in treatment programs, we need to reduce the barriers and the stigma around SUDs.We need surveillance of all SU throughout pregnancy and postnatally so that clinicians can make informed decisions about the clinical care of the mother and the developing child. A standardized measure of typical and atypical neurobehaviour should be used early in the postnatal period to identify those infants especially at risk for poor neurodevelopment. Treatment programs for SUDs during pregnancy should provide tailored, comprehensive care that considers polysubstance use and includes treatment for the co-morbidity of psychiatric problems and trauma. Finally, reducing the risks to parenting from the constellation of risk factors that are repeatedly reported in studies of prenatal drug use is paramount.
